# Prognositic factors and clinicopathologic characteristics of small gastrointestinal stromal tumor of the stomach: a retrospective analysis of 31 cases in one center

**DOI:** 10.7497/j.issn.2095-3941.2013.03.007

**Published:** 2013-09

**Authors:** Zhen Huang, Yuan Li, Hong Zhao, Jian-Jun Zhao, Jian-Qiang Cai

**Affiliations:** Department of Abdominal Surgery, Cancer Institute and Hospital, Chinese Academy of Medical Science and Peking Union Medical College, Beijing 100021, China

**Keywords:** Gastrointestinal stromal tumors, endoscopy, prognosis

## Abstract

**Objective:**

To analyze the clinicopathologic characteristics and prognostic factors of small gastrointestinal stromal tumor (GIST) of the stomach.

**Methods:**

A total of 31 small gastric GIST patients, including 10 males and 21 females, with a median age of 58 years (37-81 years), who underwent surgery at any time from 1999 to 2012 were included in this study. The clinical records of the patients were analyzed retrospectively.

**Results:**

Abdominal discomfort and pain (10 cases, 32.3%, respectively) were the two most common complaints among the patients. All patients received surgery, 11 received gastric wedge resection, 11 received subtotal gastrectomy, 5 received laparoscopic gastric wedge resection, and 4 received endoscopic submucosal dissection. No severe adverse complication was observed. A total of 29 patients (93.5%) were followed up. During the follow-up, 2 patients were found to exhibit tumor recurrence, and 1 patient had liver metastases. One patient died of tumor progression, while another died of another malignant tumor. Median progression free survival (PFS) time was 120.3 months, and median overall survival (OS) time was 130.4 months.

**Conclusion:**

Small gastric GIST has better prognosis. Surgery is the best choice for therapy. Micro-invasive procedures are safe and effective for elective patients. Tumor necrosis, tumor bleeding, and muscle invasion are potential prognostic factors of small gastric GIST.

## Introduction

Gastrointestinal stromal tumors (GIST) are the most common mesenchymal tumor in the gastrointestinal tracts, which are mostly found in the stomach^1^^,^^2^. Localized gastric stromal tumors need to be operated if the maximum diameter is over 2 cm. However, for those less than 2 cm, controversies in operating and indicating still remain. We retrospectively analyzed the clinical features of 31 patients who received surgery at the Hospital of Chinese Academy of Medical Science between March 1999 and March 2012, and discussed the clinicopathologic characteristics and relevant prognostic factors of small gastric GIST.

## Materials and methods

### Clinical data

Based on surgical and clinical data from March 1999 to March 2012, we confirmed a total of 31 cases of small gastric stromal tumor in the Hospital of Chinese Academy of Medical Science, in which there were 10 males and 21 females. The age of all the patients ranged from 37 to 81 years, with the median age of 58 years. The primary initial symptoms were abdomen discomfort and stomachache (10 cases each, 32.3%). Nine cases were diagnosed as GIST during physical examination via gastroscopy (29.0%). One case had emaciation and haematemesis. Three cases had a family history of gastric stromal tumors, which accounted for 9.8% of all cases. Nine patients were diagnosed as GIST both by computed tomography (CT) scan and gastroscope ultrasound. Eight patients were diagnosed as GIST by gastroscope ultrasound, and 9 by CT diagnosis. One case underwent magnetic resonance imaging.

### Therapy methods

Among the cases, 11 (35.5%) had subtotal gastrectomy and 11 (35.5%) had wedge resection of gastric wall. Five cases had laparoscopic wedge excision (16.1%). Four cases had laparoscopic submucosal dissection (12.9%). All cases received R0 excision. No death occurred in the perioperative period. Only 1 case who underwent wedge resection had delayed gastric emptying after operation. After symptomatic treatment, the patient was relieved and no other serious complications occurred. After operation, no imatinib adjuvant therapy was conducted. However, two cases imatinib adjuvant therapy after tumor progression (**Table 1**).

**Table 1:** Clinical data of 31 cases

Characteristics	*n* (%)
Surgery	
Subtotal gastrectomy	11 (35.5)
Gastric wedge resection	11 (35.5)
Laparoscopic gastric wedge resection	5 (16.1)
Endoscopic submucosal dissection	4 (12.9)
Site of tumor	
Cardia	4 (12.9)
Fundus	8 (25.8)
Body	13 (43.9)
Antrum	6 (19.4)
Immunohistochemical results	
CD117	27 (87.1)
CD34	27 (87.1)
S100	9 (29.0)
NIH grade	
Very low	28 (90.3)
Low	1 (3.2)
Medium	2 (6.5)
High	0
Other pathological factors	
Tumor necrosis	2 (6.5)
Tumor bleeding	2 (6.5)
Muscularis invasion	6 (19.4)

### Follow-up

We conducted follow-up visits via outpatient service review and telephone. The last follow-up visit was on March 1, 2013. Overall survival (OS) refers to the starting period of operation to the death of patients or the expiry date of follow-up visits. The progression free survival (PFS) is from the date of operation to explicit relapse and metastasis or the expiry date of follow-up visits. We conducted statistical analysis via the SPSS 19.0 software. Kaplan-Meier method was used to analyze the OS time and PFS time.

## Results

### Pathology

**Figure 1:**
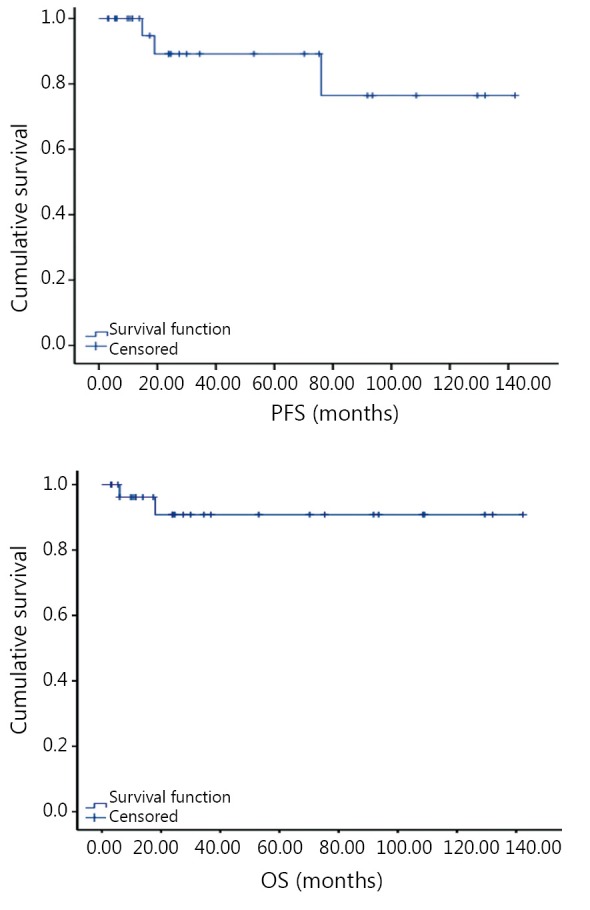
Overall survival (OS) time and progression free survival (PFS) time of 29 cases.

The tumor was mainly located in the gastric body (13 cases, 43.9%). After the operation, all cases received pathological examinations with CD117, CD34, and S100 immumohistochemical staining. CD117-negative patients received DOG1 immumohistochemical staining. All cases were diagnosed as GIST. The average maximum diameter of tumor was 1.5 cm (0.5-2.0 cm). Two cases had necrosis in the tumors (6.5%), 2 cases had internal haemorrhage (6.5%), and 6 cases had gastric mucosal involvement (19.4%). Based on the immunohistochemical results of all 31 cases, CD34 and CD117 were positively found in 27 cases each (87.1%) and positive S100 was found in 9 cases (29.0%) (**Figure 1**).

### Statistical analysis

Out of the 31 cases, 29 patients accepted follow-up visits. The rate of follow-up was 93.5%. The median follow-up time was 41.3 months (7.5-152.3 months). Two patients died during the follow-up period. One patient died because of tumor progression, and another died because of pancreatic cancer. Three cases had tumor recurrence or metastasis. Based on the classification of the National Institutes of Health (NIH), the risk factors of these 3 cases were extreme low-risk. One case had hepatic metastasis, whose pathological results showed tumor haemorrhage, necrosis, and mucosal involvement altogether. Two cases had recurrence, whose pathological results showed mucosal involvement. The average OS was estimated to be 130.4 months. The predicted value of the average PFS was 120.3 months (**Table 1**). Due to the limitation of tumor progression and death, single factor and multi-factor analyses could not be performed.

## Discussion

With the popularization of gastroscopy, especially endoscopic ultrasound, the detectable rate of gastric stromal tumors continuously increased, specifically for asymptomatic gastric stromal tumors^3^. However, there remain disputes on therapy option and prognostic factors. The malignancy of small GIST smaller than or equal to 2 cm is relatively less than larger ones, and the prognosis is better. Some even have no malignancy potential^4^. Several experts considered that small GIST can be observed closely without operation if they have no symptoms. However, with knowledge of GIST biological behaviors, we gradually realized that malignancy potential of some small GIST is higher, which needs to be positively handled. Endoscopic ultrasonography can detect malignancy potential and primary risk factors such as irregular edge, ulcer, strong echo, and heterogeneity^5^. Endoscopic ultrasonography can also determine other explicit properties via aspiration biopsy^6^. GIST needs to be operated if the patients have symptoms or poor prognostic factors appear in the endoscopic ultrasonography^7^. In this study, 22 cases needed operations because of abdominal symptoms. In 9 cases without symptoms, 4 cases showed tumor heterogeneity in the endoscopic ultrasonography and were suggested for excision. Other 5 cases were not willing to continue follow-up visits.

Surgery is the main treatment for GIST. As lymphatic metastasis is rare in gastric GIST, lymph node dissection is generally unnecessary. Wedge excision, subtotal stomach excision, and other surgical management can be selected depending on the location and size of the tumors^7^^,^^8^. With the development of laparoscopic techniques, laparoscopic surgery was generally used in the surgical treatment of GIST, besides traditional open surgery^9^^,^^10^. In this study, laparoscopic surgery was applied to 5 cases and no complications occurred after the operation. Previously it was thought that endoscopic resection was not appropriate for GIST, considering that GIST originated from the muscular layer. However, in recent years, endoscopic excision after loop ligation of the tumor has been accepted for its satisfied outcome and safety^11^^,^^12^. Laparoscopy combined with endoscopy in the treatment of GIST will further improve the outcome and safety^13^. In the clinical practice, various minimal invasive treatments should be used depending on specific situation of the tumors and the patients.

The prognosis of small GISTs is generally good, and in this study the median follow-up was 41.3 months. As the survival analysis cannot be performed because of the relatively short median PFS and OS, the estimated OS was 130.4 months and PFS was 120.3 months, which were better than that of GIST with diameter over 2 cm. According to expert consensus, most patients with small GIST are regarded with extremely low-risk or low-risk based on NIH risk factor classification^7^, therefore imatinib was unnecessary after the operation. Middle-risk patients shall at least accept imatinib for 1 year.

The influencing factors of GIST prognosis are complicated. Current studies regard tumor size and nuclear division as important indicators of the prognosis of GIST, based on which NIH risk classification standard is established. Other studies support that pathological characteristics, such as tumor necrosis and mucosa violation may be indicators of poor prognosis of GIST^14^^,^^15^. Our study showed that tumor necrosis, haemorrhage, and gastric mucosa violation may be poor prognostic factors for GIST, besides the NIH adverse factors. Further studies with more cases and longer follow-up are warranted.
